# Effect of Orem’s Self-Care Model on self-efficacy, self-management, quality of life, and HbA1c among children with type 1 diabetes mellitus in Palestine

**DOI:** 10.1186/s12909-025-08520-w

**Published:** 2026-01-05

**Authors:** Lo’ai Aburayyan, Candan Ozturk

**Affiliations:** 1 Faculty of Nursing, Palestine Polytechnic University, Hebron, PK P700 Palestine; 2 Department of Pediatric Nursing, Near East University, TRNC Mersin 10, Nicosia, PK 99138 Türkiye

**Keywords:** Orem’s Self-Care model, Type 1 diabetes mellitus, Self-efficacy, Self-management, Pediatric diabetes, HbA1c, Palestine

## Abstract

**Background:**

Type 1 diabetes mellitus (T1DM) is a growing global and national health concern, particularly among Palestinian children who face significant psychosocial, educational, and structural barriers to optimal disease management. Effective self-care requires developmentally appropriate education that enhances self-efficacy, supports family involvement, and promotes independent diabetes self-management. Orem’s Self-Care Model provides a theoretical framework for identifying self-care deficits and guiding supportive–educative interventions.

**Aim:**

To evaluate the effect of applying Orem’s Self-Care Model on self-efficacy, diabetes self-management, quality of life (QoL), and HbA1c among children with T1DM in Hebron, Palestine.

**Methods:**

A quasi-experimental pretest–posttest-controlled design was conducted among 48 children aged 8–12 years attending governmental and private pediatric diabetes clinics. Participants were allocated into intervention (*n* = 24) and control (*n* = 24) groups based on clinic grouping. The intervention comprised four structured, two-hour supportive–educative sessions grounded in Orem’s model. Standard routine care was provided to the control group. Outcomes were measured using validated Arabic versions of the Children’s Self-Efficacy Scale, PedsQL 3.0 Diabetes Module, the Diabetes Self-Management Questionnaire (DSMQ), and laboratory-recorded HbA1c. Data were analyzed using paired and independent t-tests.

**Results:**

Significant improvements were observed in the intervention group for self-efficacy, self-management, QoL, and HbA1c. HbA1c decreased from 14.0% to 10.7% (*p* = .001), compared with a nonsignificant change in the control group (13.4% to 12.5%, *p* = .306). Posttest comparisons confirmed superior outcomes in the intervention group (*p* < .05 across variables).

**Conclusion:**

Orem’s Self-Care Model significantly enhanced glycemic control and psychosocial self-care capacities among Palestinian children with T1DM. Theory-based, family-centered educational interventions represent a feasible and effective strategy for improving pediatric diabetes outcomes in resource-limited settings.

**Supplementary Information:**

The online version contains supplementary material available at 10.1186/s12909-025-08520-w.

## Introduction

Type 1 diabetes mellitus (T1DM) is a chronic autoimmune condition that continues to rise globally and represents a substantial public health burden in childhood. Recent epidemiological estimates indicate that more than 1.2 million children and adolescents worldwide are currently living with T1DM, and global prevalence is projected to increase significantly by 2045 [1]. Without effective and continuous self-management, children face heightened risks for acute complications such as diabetic ketoacidosis (DKA) and long-term microvascular and macrovascular consequences that impair daily functioning and quality of life (QoL) [2, 3]. Consequently, pediatric diabetes care increasingly emphasizes structured education, development of self-efficacy, and family-centered support as essential components for optimizing glycemic control and preventing complications.

In the Palestinian context, the burden of childhood diabetes is particularly challenging. More than 3,600 Palestinian children and adolescents are living with T1DM, with disproportionate clustering in governorates such as Hebron and Jenin [4]. Beyond limited health-system resources, many children experience difficulty adhering to diabetes self-management routines due to financial constraints, psychosocial pressures, inadequate access to specialized diabetes services, and variability in caregiver knowledge [4, 5]. A growing body of qualitative evidence from Palestine reveals that children and adolescents often struggle with fear, emotional distress, parental overprotection, and disruptions to school and social life—factors that directly undermine diabetes management.

Harazneh, Malak, and Ayed (2024) found that Palestinian adolescents experience a complex, nonlinear process of adapting to T1DM, characterized by fluctuating emotions, changing family dynamics, and evolving identity challenges [6]. These psychosocial burdens influence self-management behaviors and highlight the need for structured, developmentally appropriate interventions. Complementary evidence from Nazzal et al. (2025) demonstrates that Palestinian youth employ coping strategies shaped by cultural norms, family relationships, and healthcare constraints, further emphasizing the importance of supportive educational frameworks rooted in local realities [7]. These qualitative findings collectively indicate that the management of T1DM in Palestine extends beyond biomedical treatment and requires an integrative approach that simultaneously addresses emotional well-being, self-efficacy, and family involvement.

Furthermore, Palestinian clinical research underscores the consequences of inconsistent diabetes education. Younis et al. (2025) showed that applying standardized clinical guidelines in emergency departments significantly improved outcomes in pediatric DKA cases, illustrating how structured, evidence-based interventions can directly influence clinical trajectories [8]. Such findings reinforce the need for theory-driven approaches that can be applied not only in acute settings but also in routine self-management.

Orem’s Self-Care Deficit Nursing Theory offers a valuable framework for addressing these gaps. The theory proposes that individuals require nursing support when they cannot independently meet their self-care needs due to developmental or health-related limitations [9, 10]. Its “supportive–educative” system is particularly suitable for children with T1DM, who depend on both parental guidance and structured skill-building to achieve autonomy in diabetes management. By aligning educational content with children’s developmental level and family context, Orem’s model can strengthen self-efficacy, enhance self-management behaviors, and support psychosocial adaptation—critical components identified as lacking in Palestinian studies [6, 7, 11, 12].

Despite the relevance of Orem’s theory, there is a significant research gap in Palestine regarding theory-based pediatric diabetes interventions. Existing programs often lack structured frameworks, do not fully involve caregivers, or are not tailored to children’s developmental and psychosocial needs. Few studies have examined comprehensive outcomes such as self-efficacy, self-management behaviors, QoL, and HbA1c in an integrated model. Addressing this gap is essential for developing culturally congruent interventions capable of improving long-term diabetes outcomes in Palestinian children.

Therefore, this study aims to evaluate the effect of applying Orem’s Self-Care Model on self-efficacy, diabetes self-management, QoL, and HbA1c among children aged 8–12 years with T1DM in Hebron, Palestine. By grounding the intervention in Orem’s supportive–educative system and integrating the family as active participants, this study provides evidence for a theoretically robust, context-specific, and developmentally appropriate approach to pediatric diabetes management.

## Methods

### Study design

This study employed a quasi-experimental pretest–posttest controlled design to evaluate the effect of applying Orem’s Self-Care Model on self-efficacy, diabetes self-management, quality of life (QoL), and HbA1c among children with type 1 diabetes mellitus (T1DM). The study was conducted between March and July 2025 in pediatric diabetes and endocrine clinics in Hebron, Palestine.

### Study setting

Data were collected from four clinics, two governmental and two private, to ensure representation of different healthcare delivery environments. Two clinics served as the intervention sites and two as the control sites. This clinic-based grouping minimized contamination between groups, consistent with quasi-experimental methodology.

### Sampling method and eligibility criteria

A purposive sampling strategy was used to recruit eligible participants. Children were included if they:

- Were aged 8–12 years.
- Had a confirmed diagnosis of T1DM for at least 6 months.
- Were followed routinely at one of the participating clinics.
- Were able to participate in educational sessions with a caregiver.
- Had parental consent and child assent.

Children with cognitive impairments, comorbid chronic diseases, or recent hospitalization for severe complications were excluded.

### Sample size determination

The sample size was calculated using the difference-in-means formula and informed by a previous study reporting moderate effect sizes in diabetes self-management interventions. After adjusting for an anticipated 20% attrition rate, a total of 48 children were required, 24 in the intervention group and 24 in the control group.

### Group allocation

Because randomization was not feasible in this real-world clinical context, participants were assigned according to the natural grouping of their clinic. From each clinic type (governmental and private), **1**2 children were allocated to the intervention group and 12 to the control group, ensuring balanced representation and reducing setting-related bias.

### Intervention: Orem’s Self-Care model–based program

The intervention was grounded in Orem’s Self-Care Deficit Nursing Theory, specifically the supportive–educative system, aiming to enhance children’s self-care agency and parental involvement.

### Assessment phase

Before beginning the intervention, individualized self-care deficits were identified through:

- Child and caregiver interviews.
- Observation.
- Review of medical records.
- Nurse educator assessment.

These assessments guided the tailoring of educational content.

### Structure of the intervention

The program consisted of four structured training sessions, each lasting approximately 2 h, delivered through a combination of online meetings (Google Meet) and face-to-face sessions. Both children and caregivers participated in all sessions.

The intervention emphasized general self-care (nutrition, hygiene, stress management), developmental needs, and self-management during illness (insulin use, monitoring, clinic visits).

### Fidelity measures

- A single trained pediatric nurse educator delivered all sessions.
- Standardized lesson plans and checklists were used.
- Attendance was recorded for both children and caregivers.

### Control group

Participants in the control clinics received standard routine care, including general diabetes education and follow-up per clinic protocol. They did not receive structured sessions based on Orem’s theory.

### Data collection instruments

Four validated instruments were used in this study:

1. Structured Sociodemographic Questionnaire
  Developed by the researchers to collect demographic and clinical information.
2. Children’s Self-Efficacy Questionnaire
  Originally developed by Muris (2001). Higher scores indicate greater self-efficacy.
3. Pediatric Quality of Life Inventory (PedsQL) 3.0 Diabetes Module
  Measures diabetes-specific QoL across multiple domains.
4. Diabetes Self-Management Questionnaire (DSMQ)
  Adapted from previous self-care educational intervention studies.

### Translation and cultural adaptation

All instruments were administered in Arabic. Tools underwent:

- Forward translation by two bilingual experts.
- Back-translation by an independent translator.
- Expert panel review.
- Pilot testing with 10 children (excluded from the main sample).

### Psychometric properties

Cronbach’s alpha values for the current sample:

- Self-efficacy scale: α = 0.88.
- PedsQL Diabetes Module: α = 0.91.
- DSMQ: α = 0.86.

These values demonstrate strong internal consistency.

### Outcome measures

Primary outcomes:

- Self-efficacy.
- Diabetes self-management.
- Quality of life.
- HbA1c levels (obtained from clinic laboratory records).

Assessments occurred at baseline (pretest) and 8 weeks after the intervention (posttest).

### Statistical analysis

Data were analyzed using SPSS version 23.

### Pre-analysis procedures

- Data were screened for missing values, outliers, and normality using the Shapiro–Wilk test and histograms.
- No cases required imputation or exclusion.

### Analytical strategy

- Descriptive statistics (means, SDs, frequencies) summarized participant characteristics.
- Independent t-tests compared baseline equivalence between groups.
- Paired t-tests assessed within-group changes from pretest to posttest.
- Independent t-tests assessed posttest differences between groups.
- The significance level was set at *p* < .05.

## Results

### Participant characteristics

A total of 48 children with T1DM participated in the study (24 intervention, 24 control). Table 1 presents the sociodemographic characteristics of the sample. The mean age of participants was 10.10 years (SD = 1.30). Most questionnaires were completed by mothers (50%), and the majority of participating children were female (60.4%). Approximately 83.3% of children lived with both parents. Parental education levels varied, with 37.5% of fathers and 27.1% of mothers reporting completion of high school.

**Table 1:** Structure of the intervention

Session	Topics Covered	Activities	Mode & Materials
**1**	Introduction to diabetes (definition, types, symptoms), complications, risk factors, eye and foot care	Discussion, demonstration, Q&A	Online + face-to-face; PowerPoint, brochures
**2**	Diabetes control, blood glucose monitoring using a glucometer	Demonstration, guided practice	Online + face-to-face; PowerPoint, videos, leaflets
**3**	Nutrition and exercise for diabetic children	Interactive discussion, problem-solving exercises	Online + face-to-face; educational videos, brochures
**4**	Consolidation of self-care skills, educational materials, and video sessions	Review, practice, and distribution of take-home materials	Online + face-to-face; pamphlets, educational CD

According to Table 2, the demographic profile shows that most participating children were school-aged females living with both parents, with mothers being the primary respondents. The high percentage of parental unemployment—particularly among mothers—may indicate socioeconomic barriers that could affect diabetes self-management and access to educational resources.

**Table 2:** Sociodemographic characteristics of participants (*N* = 48)**

Variable	Category	*n* (%)	M (SD)
Age (years)	—	—	10.10 (1.30)
Who completed the questionnaire?	Mother	24 (50.0)	—
	Father	6 (12.5)	
	Sibling	5 (10.4)	
	Child	13 (27.1)	
Gender	Male	19 (39.6)	—
	Female	29 (60.4)	
Grade level	4th grade	20 (41.7)	—
	5th grade	6 (12.5)	
	6th grade	10 (20.8)	
	7th grade	8 (16.7)	
	8th grade	4 (8.3)	
Living with parents	Yes	40 (83.3)	—
	No	8 (16.7)	
Father’s education	< High school	12 (25.0)	—
	High school	18 (37.5)	
	Bachelor	16 (33.3)	
	Master+	2 (4.2)	
Father’s occupation	Unemployed	23 (47.9)	—
	Part-time	8 (16.7)	
	Full-time	17 (35.4)	
Mother’s education	< High school	9 (18.8)	—
	High school	13 (27.1)	
	Diploma	14 (29.2)	
	Bachelor	10 (20.8)	
	Master+	2 (4.2)	
Mother’s occupation	Unemployed	33 (68.8)	—
	Part-time	2 (4.2)	
	Full-time	13 (27.1)	

### Baseline comparisons between groups

An independent-samples t-test showed no statistically significant baseline differences between the intervention and control groups for self-efficacy, QoL, or diabetes self-management (*p* > .05), confirming initial group equivalence.

According to Table 3, both groups were comparable at baseline on all key outcome measures. This supports the internal validity of the quasi-experimental design by demonstrating that any post-intervention differences are unlikely due to pre-existing variability.

**Table 3:** Baseline differences between intervention and control groups (*N* = 48)**

Outcome	Control M (SD)	Intervention M (SD)	Levene’s Test *p*	t-test *p*
Self-efficacy	3.0 (0.6)	3.0 (0.6)	0.927	0.931
Quality of life	61.30 (12.30)	57.20 (17.00)	0.101	0.336
Diabetes self-management	4.50 (1.30)	4.70 (0.90)	0.089	0.591

### HbA1c differences between groups at posttest

At posttest, the intervention group exhibited significantly lower HbA1c levels compared with the control group (*p* = .026), indicating clinically meaningful improvement following the Orem-based intervention.

According to Table 4. Children in the intervention group achieved significantly better glycemic control, reflected by reduced HbA1c levels. The large SD in the control group suggests persistent variability and poorer regulation compared with the structured intervention group.

**Table 4:** Posttest HbA1c comparison between groups (*N* = 48)**

Outcome	Control M (SD)	Intervention M (SD)	t	*p*
HbA1c (%)	12.50 (3.60)	10.70 (1.30)	–2.29	0.026

### Within-group changes in HbA1c

#### Intervention group

A paired-samples t-test revealed a significant decrease in HbA1c from pretest (M = 14.00) to posttest (M = 10.70), *p* = .001.

According to Table 5, the intervention group showed a substantial and statistically significant improvement in glycemic control. This reduction of more than 3 HbA1c points is clinically meaningful and aligns with the goals of self-care–centered diabetes management.

**Table 5:** Pretest–Posttest HbA1c changes: intervention group (*n* = 24)**

Time	M (SD)	t	*p*
Pretest	14.00 (2.30)	—	—
Posttest	10.70 (1.30)	6.05	0.001

#### Control group

The control group did not show a significant change from pretest to posttest (*p* = .306).

According to Table 6, the absence of meaningful HbA1c improvement in the control group suggests that routine diabetic care alone was insufficient to produce significant metabolic changes. This highlights the added value of structured, theory-based self-care interventions.

**Table 6:** Pretest–Posttest HbA1c changes: control group (*n* = 24)**

Time	M (SD)	t	*p*
Pretest	13.40 (3.10)	—	—
Posttest	12.50 (3.60)	1.05	0.306

## Discussion

The present study examined the effectiveness of an Orem-based supportive–educative intervention on glycemic control, self-efficacy, diabetes self-management, and quality of life among children with T1DM in Hebron. The results demonstrated significant improvements in the intervention group compared with routine care, particularly in HbA1c reduction. These findings reinforce global and regional evidence showing that structured, theory-driven diabetes education enhances both clinical and psychosocial outcomes in pediatric populations.

The significant reduction in HbA1c among the intervention group aligns with international evidence that self-management education improves metabolic control in children with T1DM. Elhawy et al. demonstrated that tailored caregiver education improved glycemic markers and adherence among Egyptian children with T1DM [9]. Similar findings were reported by Johnson et al., who noted that structured diabetes education led to better HbA1c trajectories over time in large pediatric cohorts [10]. Randomized controlled trials in Europe and North America have consistently shown that educational programs emphasizing self-care, glucose monitoring, and behavioral skill-building produce clinically meaningful reductions in HbA1c levels [11–13].

Importantly, the present study supports Palestinian evidence describing the psychosocial challenges experienced by children with T1DM. Qualitative studies by Harazneh et al. [6] and Nazzal et al. [7] illustrate how emotional distress, disrupted routines, and family dynamics undermine children’s self-care abilities. Orem’s model is well-suited to address these challenges, as it emphasizes identifying self-care deficits and building patient and caregiver capacity through supportive–educative actions [14]. The improved outcomes observed here suggest that the intervention helped children gain independence, confidence, and clarity in their diabetes-related responsibilities.

The findings also parallel outcomes in interventions from other Middle Eastern and low-resource settings. Kamal and Fawzy found that structured pediatric diabetes education improved self-management and metabolic control among Egyptian children [15]. A study from Jordan by Kaddoura and colleagues reported that Orem-based nursing care improved children’s functional outcomes and caregiver involvement [16]. In Iran, Karimi et al. found that self-care education significantly enhanced diabetes knowledge, self-efficacy, and glycemic control in school-aged children [17]. A Saudi Arabian study similarly reported improved metabolic outcomes after implementing a family-centered diabetes education model [18]. The present findings add to this growing regional evidence base by demonstrating the value of theory-guided interventions in Palestine, where structural and psychosocial barriers are particularly pronounced.

The improvements observed in HbA1c can also be explained through behavioral and theoretical mechanisms. Orem’s model posits that individuals can develop self-care agency when provided with structured support tailored to their developmental needs [14]. Research shows that increases in self-efficacy directly mediate better glycemic control and treatment adherence in children [19, 20]. Whittemore and Grey’s meta-analyses demonstrate that interventions targeting coping skills, diabetes-related confidence, and parent–child collaboration substantially improve self-management behaviors [21, 22]. In the current study, repeated demonstrations, interactive activities, and parental involvement likely strengthened children’s procedural skills (e.g., insulin administration), self-regulatory abilities (e.g., glucose monitoring), and confidence in illness management.

Evidence also indicates that quality of life improves when glycemic control stabilizes and self-care demands become more manageable. Multiple studies have shown that diabetes-specific QoL is closely linked with children’s perceived competence and emotional adjustment [23–25]. Although QoL was not the most dramatic clinical change observed in this study, the direction of improvement aligns with established models of pediatric adaptation to chronic illness.

The findings further support the importance of standardized and systematic diabetes care in Palestine. As shown in Younis et al.’s study on pediatric DKA, implementing clinical guidelines improves outcomes even in resource-constrained environments [8]. When combined with a theoretical framework such as Orem’s, structured education becomes even more powerful, offering a pathway toward sustainable improvements in chronic disease self-management.

However, several limitations must be acknowledged. The quasi-experimental design introduces potential selection bias, although baseline equivalence was confirmed. The study was also limited to one city, with a modest sample size and a short follow-up duration, limiting generalizability. Furthermore, some data were caregiver-reported, which may introduce social desirability bias. Despite these limitations, the study demonstrates strong internal consistency, rigorous cultural adaptation of tools, and clear intervention fidelity, supporting the validity of the findings.

In summary, this study reinforces the effectiveness of Orem’s Self-Care Model as a culturally appropriate and clinically meaningful framework for pediatric diabetes care in Palestine. By enhancing children’s self-efficacy, strengthening family engagement, and improving self-management behaviors, the model contributes to better glycemic outcomes and lays the foundation for scalable interventions across the Palestinian healthcare system.

### Implication

This study provides compelling evidence that applying Orem’s Self-Care Model can substantially enhance diabetes-related outcomes among Palestinian children with type 1 diabetes mellitus. The supportive–educative intervention improved children’s self-efficacy, strengthened self-management behaviors, and resulted in a clinically meaningful reduction in HbA1c levels compared with routine care. By addressing self-care deficits and actively involving caregivers, the model promoted greater autonomy and confidence in daily diabetes tasks. These findings underscore the value of theory-based, family-centered education within pediatric chronic disease management and highlight its potential to improve long-term health outcomes in resource-limited healthcare settings.

## Conclusion

This study demonstrated that applying Orem’s Self-Care Model significantly improved glycemic control, self-efficacy, diabetes self-management, and quality of life among Palestinian children with type 1 diabetes mellitus. The structured supportive–educative intervention effectively addressed children’s self-care deficits, strengthened caregiver involvement, and facilitated greater autonomy in daily diabetes tasks—resulting in a clinically meaningful reduction in HbA1c levels compared with routine care. These findings provide robust evidence that theory-based, family-centered educational programs are both feasible and beneficial in resource-limited settings and should be incorporated into pediatric diabetes services to optimize long-term health outcomes for children living with T1DM.

## Supplementary Information

Supplementary Material 1.

Supplementary Material 2.

## Data Availability

The datasets used and/or analyzed during the current study are available from the corresponding author on reasonable request.
