# Supplementary material for: Effect of Orem’s Self-Care Model on self-efficacy, self-management, quality of life, and HbA1c among children with type 1 diabetes mellitus in Palestine
Source: BMC Med Educ. 2026 Jan 5;26:539. doi: 10.1186/s12909-025-08520-w (PMC13041130; doi:10.1186/s12909-025-08520-w)
Supplement: Supplementary file 1 — Supplementary Material 1. [file 12909_2025_8520_MOESM1_ESM.pdf]

نموذج موافقة مستنيرة على المشاركة في بحث علمي

عزيزي الطالب\ة انت مدعو للمشاركة في بحث علمي يقوم به الدكتورلؤي أبوريان من كلية التمريض في جامعة الشرق الادنى. نحن نتطلع الى الافادة العلمية من خلال دراسة (تأثير نموذج أوريم للرعاية الذاتية على فعالية الذات، وإدارة مرض السكري، وجودة الحياة، ومستوى الهيموغلوبين السكري لدى الأطفال المصابين بداء السكري من النوع الأول)، ويهدف هذا البحث إلى تقييم تاثيرالبرنامج التعليمي الخاص ب نموذج أوريم للرعاية الذاتية على فعالية الذات، وإدارة مرض السكري، وجودة الحياة، ومستوى الهيموغلوبين السكري لدى الأطفال المصابين بداء السكري من النوع الأول.

المشاركة في هذا البحث هي اختيارية محضة، حيث تم اختيارك بشكل عشوائي من بين الطلاب الاخرين ضمن نفس المواصفات. كما انه لا توجد اي مخاطر جسدية او نفسية متعلقة بالمشاركة، مع ضمان السرية والخصوصية التامة للمشاركين في هذه الدراسة، مع التعهد بعدم استخدام اي معلومات شخصية او طبية لغير أغراض البحث العلمي فقط، مؤكدين على ان الاسماء غير مطلوبة وتبقى سرية. كما ان المشاركة في هذا البحث تعود بالفائدة عليك وعلى جميع الطلاب وعلى المجتمع ككل، مع عدم التعهد باي منافع شخصية مباشرة للمشاركين في البحث.

إذا قررت المشاركة في هذه الدراسة سوف يقوم شخص مهني بمقابلتك واعطائك استبيان لملئه في مدة لا تتجاوز الربع ساعة، ونؤكد على انه يبقى لك كامل الحق في المشاركة في الدراسة او رفضها او الانسحاب منها في اي وقت تشاء. كما نؤكد ان لجنة خاصة في الجامعة قامت بمراجعة هذا البحث العلمي واعطاء الموافقة عليه ضمن المعايير العلمية والبحثية الوطنية والعالمية المعمول بها في الجامعة.

بمجرد موافقتك على تعبئه الاستبيان المرفق، يعتبر ذلك بمثابه موافقة ضمنية. وفي حال وجود اي استفسار حالي او لاحق يمكن الاتصال مع الشخص القائم على البحث في اي وقت على العنوان التالي:

الاسم: لؤي أبوريان

المهنة: تمريض

هاتف: 00972599339140

Email: loai@ppu.edu.ps

انت في طور الموافقة على المشاركة في هذا البحث، وتوقيعك فيما يلي يعني هذه الموافقة وكذلك اقرارك بقراءة ما تقدم في هذا النموذج.

المشارك: ____________________ التوقيع: ____________________

ولي الامر: ____________________ التوقيع: ____________________

التاريخ: ____________
