# Supplementary material for: Effect of Orem’s Self-Care Model on self-efficacy, self-management, quality of life, and HbA1c among children with type 1 diabetes mellitus in Palestine
Source: BMC Med Educ. 2026 Jan 5;26:539. doi: 10.1186/s12909-025-08520-w (PMC13041130; doi:10.1186/s12909-025-08520-w)
Supplement: Supplementary file 2 — Supplementary Material 2. [file 12909_2025_8520_MOESM2_ESM.docx]

**NEAR EAST UNIVERSITY**

**INSTITUTE OF GRADUATE STUDIES**

**DEPARTMENT OF Pediatric Nursing**

**Effect of Orem's self-care model and self-care management on the quality of life and HbA1c levels in children with diabetes type 1**

**Doctoral of Nursing**

**Lo’ai Abu-Rayyan**

Greetings and Regards;

The information of this questionnaire is completely confidential. So there is no need to write your name. Please answer the questions carefully and let us know your valuable comments. We hope that the results of this questionnaire can be effective in improving your health.

You are free to participate or not to participate in this research.

**Demographic Data Questionnaire (English Version)**

**Date:** …………………………… **Participant Code Number:** ………………………………

**Demographic Data Questionnaire**

- Who is answering the general questions of this questionnaire?
  - The Participant ☐ His Father ☐ His Mother ☐ Other (Please specify )
- Please answer each question as accurately as possible by circling the correct answer or filling in the space provided.

1. What is your age?
2. What is your gender? ☐ Female ☐ Male
3. Are you currently a student? ☐ Yes ☐ No
   - If (Yes), what is your grade?
4. Parents marital status: ☐ Married ☐ Divorced
5. Do you live with both of them? ☐ Yes ☐ No
   - If (No), what is the reason? ☐Death ☐Divorced ☐Father’s travel abroad
6. What is the highest level of education your father has completed?

- Less than high school degree
- High school degree or equivalent
- College degree
- Bachelor degree
- Master degree
- Doctoral degree

1. What is the highest level of education your mother has completed?

- Less than high school degree
- High school degree or equivalent
- College degree
- Bachelor degree
- Master degree
- Doctoral degree

1. What is your father employment status?

- Unemployed
- Part-time
- Full-time

1. What is your mother employment status?

- Unemployed
- Part-time
- Full-time

1. Duration of diabetes (years):
2. Age at diagnosis:
3. result of the last A1C : _______
4. Does anyone in your family have diabetes?

🞏 Yes

🞏 No

1. If the answer is (yes), please mark (√) the person:

- Father
- Mother
- Father and Mother
- Sister
- Brother

1. I follow up a diabetes doctor at the:

- Private clinic.
- Governmental clinic

1. Do you suffer from any of the following diseases in addition to DM?

- I do not suffer from any other diseases.
- Cystic fibrosis
- Asthma
- Heart and arterial disease
- Kidney disease
- Immune disease
- Eating disorders
- Depression
- I don't know
- Other diseases_____________

1. Length in centimeters ___________
2. Weight _______________ KG
3. How do you take your insulin dose?

- (subcutaneous injections)
- **PO**

1. If the answer is (daily insulin injections), how many injections are there per day?

- Once daily
- Twice daily
- 3 times
- 4 times
- 5 TIMES or more

1. How many times is blood sugar measured daily?

- Once daily
- Twice daily
- 3 times
- 4 times
- 5 times
- 6 and more

1. Do you have health insurance?

- Yes
- No

1. How many times have you been hospitalized for diabetes in the past year?

- Once
- Twice
- 3 times
- 4 times
- 5 times
- 6 and more

1. How many episodes of hypoglycemia did you experience last month?

- Once
- Twice
- 3 times
- 4 times
- 5 times
- 6 times
- 7 times
- 8 times
- 9 times
- 10 and more
- Children's Self-Efficacy Questionnaire

1. How helpful are teachers when you get stuck in school work?

- Excellent (5)
- Very good (4)
- Acceptable (3)
- Very little (2)
- Not at all (1)

1. How well can you express your opinions when your classmates disagree with you?

- Excellent (5)
- Very good (4)
- Acceptable (3)
- Very little (2)
- Not at all (1)

1. How well do you cheer yourself up when an unpleasant event occurs?

- Excellent (5)
- Very good (4)
- Acceptable (3)
- Very little (2)
- Not at all (1)

1. How well can you concentrate on studying when there are other fun things to do?

- Excellent (5)
- Very good (4)
- Acceptable (3)
- Very little (2)
- Not at all (1)

1. How successful are you at becoming calm again when you're so afraid?

- Excellent (5)
- Very good (4)
- Acceptable (3)
- Very little (2)
- Not at all (1)

1. How well are you able to make friends with other children?

- Excellent (5)
- Very good (4)
- Acceptable (3)
- Very little (2)
- Not at all (1)

1. How well do you study a class for a test?

- Excellent (5)
- Very good (4)
- Acceptable (3)
- Very little (2)
- Not at all (1)

1. How well can you carry on a conversation with an unfamiliar person?

- Excellent (5)
- Very good (4)
- Acceptable (3)
- Very little (2)
- Not at all (1)

1. How much can you prevent becoming nervous?

- Excellent (5)
- Very good (4)
- Acceptable (3)
- Very little (2)
- Not at all (1)

1. How well do you do in finishing all of your schoolwork each day?

- Excellent (5)
- Very good (4)
- Acceptable (3)
- Very little (2)
- Not at all (1)

1. How well can you work in harmony with your classmates?

- Excellent (5)
- Very good (4)
- Acceptable (3)
- Very little (2)
- Not at all (1)

1. How much can you control your emotions?

- Excellent (5)
- Very good (4)
- Acceptable (3)
- Very little (2)
- Not at all (1)

1. How well can you pay attention during each chapter?

- Excellent (5)
- Very good (4)
- Acceptable (3)
- Very little (2)
- Not at all (1)

1. How well can you tell other children that they are doing something you don't like?

- Excellent (5)
- Very good (4)
- Acceptable (3)
- Very little (2)
- Not at all (1)

1. How well can you tell a funny event to a group of children?

- Excellent (5)
- Very good (4)
- Acceptable (3)
- Very little (2)
- Not at all (1)

1. How often can you tell a friend that you are not feeling well?

- Excellent (5)
- Very good (4)
- Acceptable (3)
- Very little (2)
- Not at all (1)

1. How successful have you been in pleasing your parents with your schoolwork?

- Excellent (5)
- Very good (4)
- Acceptable (3)
- Very little (2)
- Not at all (1)

1. How successful are you at staying friends with other kids?

- Excellent (5)
- Very good (4)
- Acceptable (3)
- Very little (2)
- Not at all (1)

1. How successful are you at suppressing unpleasant thoughts?

- Excellent (5)
- Very good (4)
- Acceptable (3)
- Very little (2)
- Not at all (1)

1. How well did you pass the test?

- Excellent (5)
- Very good (4)
- Acceptable (3)
- Very little (2)
- Not at all (1)

1. How successful are you at preventing conflicts with other children?

- Excellent (5)
- Very good (4)
- Acceptable (3)
- Very little (2)
- Not at all (1)

1. How successful are you at not worrying about things that might happen?

- Excellent (5)
- Very good (4)
- Acceptable (3)
- Very little (2)
- Not at all (1)

1. I feel hungry

- Never (0)
- Rarely (1)
- Sometimes (2)
- Often (3)
- Always (4)

1. I feel thirsty

- Never (0)
- Rarely (1)
- Sometimes (2)
- Often (3)
- Always (4)

1. I have to go to the bathroom very often

- Never (0)
- Rarely (1)
- Sometimes (2)
- Often (3)
- Always (4)

1. I have colic

- Never (0)
- Rarely (1)
- Sometimes (2)
- Often (3)
- Always (4)

1. I have a headache

- Never (0)
- Rarely (1)
- Sometimes (2)
- Often (3)
- Always (4)

1. Blood sugar level decreases

- Never (0)
- Rarely (1)
- Sometimes (2)
- Often (3)
- Always (4)

1. I feel tired

- Never (0)
- Rarely (1)
- Sometimes (2)
- Often (3)
- Always (4)

1. I feel shivering

- Never (0)
- Rarely (1)
- Sometimes (2)
- Often (3)
- Always (4)

1. I'm drenched in sweat

- Never (0)
- Rarely (1)
- Sometimes (2)
- Often (3)
- Always (4)

1. I don't sleep well

- Never (0)
- Rarely (1)
- Sometimes (2)
- Often (3)
- Always (4)

1. I became agitated quickly

- Never (0)
- Rarely (1)
- Sometimes (2)
- Often (3)
- Always (4)

1. I feel pain when I prick my finger or give insulin injections

- Never (0)
- Rarely (1)
- Sometimes (2)
- Often (3)
- Always (4)

1. I'm embarrassed by my diabetes

- Never (0)
- Rarely (1)
- Sometimes (2)
- Often (3)
- Always (4)

1. My parents and I argue about diabetes care

- Never (0)
- Rarely (1)
- Sometimes (2)
- Often (3)
- Always (4)

1. It is difficult for me to stick to a care plan for an illness

- Never (0)
- Rarely (1)
- Sometimes (2)
- Often (3)
- Always (4)

1. It is difficult to perform a blood sugar test

- Never (0)
- Rarely (1)
- Sometimes (2)
- Often (3)
- Always (4)

1. It is difficult to take insulin injections

- Never (0)
- Rarely (1)
- Sometimes (2)
- Often (3)
- Always (4)

1. It is difficult for me to do physical activities

- Never (0)
- Rarely (1)
- Sometimes (2)
- Often (3)
- Always (4)

1. It is difficult for me to monitor my carbohydrate intake or alternatives

- Never (0)
- Rarely (1)
- Sometimes (2)
- Often (3)
- Always (4)

1. It's hard for me to wear a diabetes data bracelet

- Never (0)
- Rarely (1)
- Sometimes (2)
- Often (3)
- Always (4)

1. It is difficult to consume fast-acting carbohydrates

- Never (0)
- Rarely (1)
- Sometimes (2)
- Often (3)
- Always (4)

1. It's hard for me to snack

- Never (0)
- Rarely (1)
- Sometimes (2)
- Often (3)
- Always (4)

1. I worry that “my blood sugar will drop”

- Never (0)
- Rarely (1)
- Sometimes (2)
- Often (3)
- Always (4)

1. I worry about whether the medical treatments I receive are working or not

- Never (0)
- Rarely (1)
- Sometimes (2)
- Often (3)
- Always (4)

1. I worry about long-term complications from diabetes

- Never (0)
- Rarely (1)
- Sometimes (2)
- Often (3)
- Always (4)

1. It's hard for me to tell the doctors and nurses how I feel

- Never (0)
- Rarely (1)
- Sometimes (2)
- Often (3)
- Always (4)

1. It is difficult for me to ask questions to doctors and nurse

- Never (0)
- Rarely (1)
- Sometimes (2)
- Often (3)
- Always (4)

1. It is difficult for me to explain my illness to others

- Never (0)
- Rarely (1)
- Sometimes (2)
- Often (3)
- Always (4)

**(Appendix 2 (**

Greetings and Regards;

Thank you for your time, the following statements describe self-care activities related to your diabetes. Thinking about your self-care over the last 8 weeks, please specify the extent to which each statement applies to you.

The information of this questionnaire is completely confidential. So there is no need to write your name. Please answer the questions carefully and let us know your valuable comments. We hope that the results of this questionnaire can be effective in improving your health.

You are free to participate or not to participate in this research.

**Diabetes Self-Management Questionnaire (DSMQ)**

| Questions | | Applies to  me very  much  (3) | Applies to me  to a consider-able  degree  (2) | Applies to  me to some  degree  (1) | Does not  apply to  me  (0) |
| --- | --- | --- | --- | --- | --- |
| 1 | I check my blood sugar levels with care and attention.  🞏 Blood sugar measurement is not required as a part of my treatment. |  |  |  |  |
| 2 | The food I choose to eat makes it easy to achieve optimal blood sugar levels. |  |  |  |  |
| 3 | I keep all doctors’ appointments recommended for my diabetes treatment. |  |  |  |  |
| 4 | I take my diabetes medication (e. g. insulin, tablets) as prescribed. / Diabetes medication / insulin is not required as a part of my treatment. |  |  |  |  |
| 5 | Occasionally I eat lots of sweets or other foods rich in carbohydrates. |  |  |  |  |
| 6 | I record my blood sugar levels regularly (or analyses the value chart with my blood glucose meter)./ Blood sugar measurement is not required as a part of my treatment. |  |  |  |  |
| 7 | I tend to avoid diabetes-related doctors’ appointments. |  |  |  |  |
| 8 | I do regular physical activity to achieve optimal blood sugar levels. |  |  |  |  |
| 9 | I strictly follow the dietary recommendations given by my doctor or diabetes specialist. |  |  |  |  |
| 10 | I do not check my blood sugar levels frequently enough as would be required for achieving good blood glucose control./ Blood sugar measurement is not required as a part of my treatment. |  |  |  |  |
| 11 | I avoid physical activity, although it would improve my diabetes. |  |  |  |  |
| 12 | I tend to forget to take or skip my diabetes medication (e. g. insulin, tablets)./ Diabetes medication / insulin is not required as a part of my treatment. |  |  |  |  |
| 13 | Sometimes I have real ‘food binges’ (not triggered by hypoglycemia). |  |  |  |  |
| 14 | Regarding my diabetes care, I should see my medical practitioner(s) more often. |  |  |  |  |
| 15 | I tend to skip planned physical activity. |  |  |  |  |
| 16 | My diabetes self-care is poor. |  |  |  |  |
